# Supplementary material for: Time‑resolved multi-omic analysis of paclitaxel exposure in human iPSC‑derived sensory neurons unveils mechanisms of chemotherapy‑induced peripheral neuropathy
Source: Cell Death Dis. 2026 Feb 10;17(1):211. doi: 10.1038/s41419-026-08445-2 (PMC12921266; doi:10.1038/s41419-026-08445-2)
Supplement: Supplementary file 15 — Dataset 1. [file 41419_2026_8445_MOESM15_ESM.zip › CDD_Open_Data/04_RNA_Seq/Timeline_panel_plots_etc_2023-01-23_1609.zip!/Panel_Plots_etc/02_contrasts_tmod_panel_plot_tmod_pval_filter_0.01_AUC0.7_L2FC1.pdf]

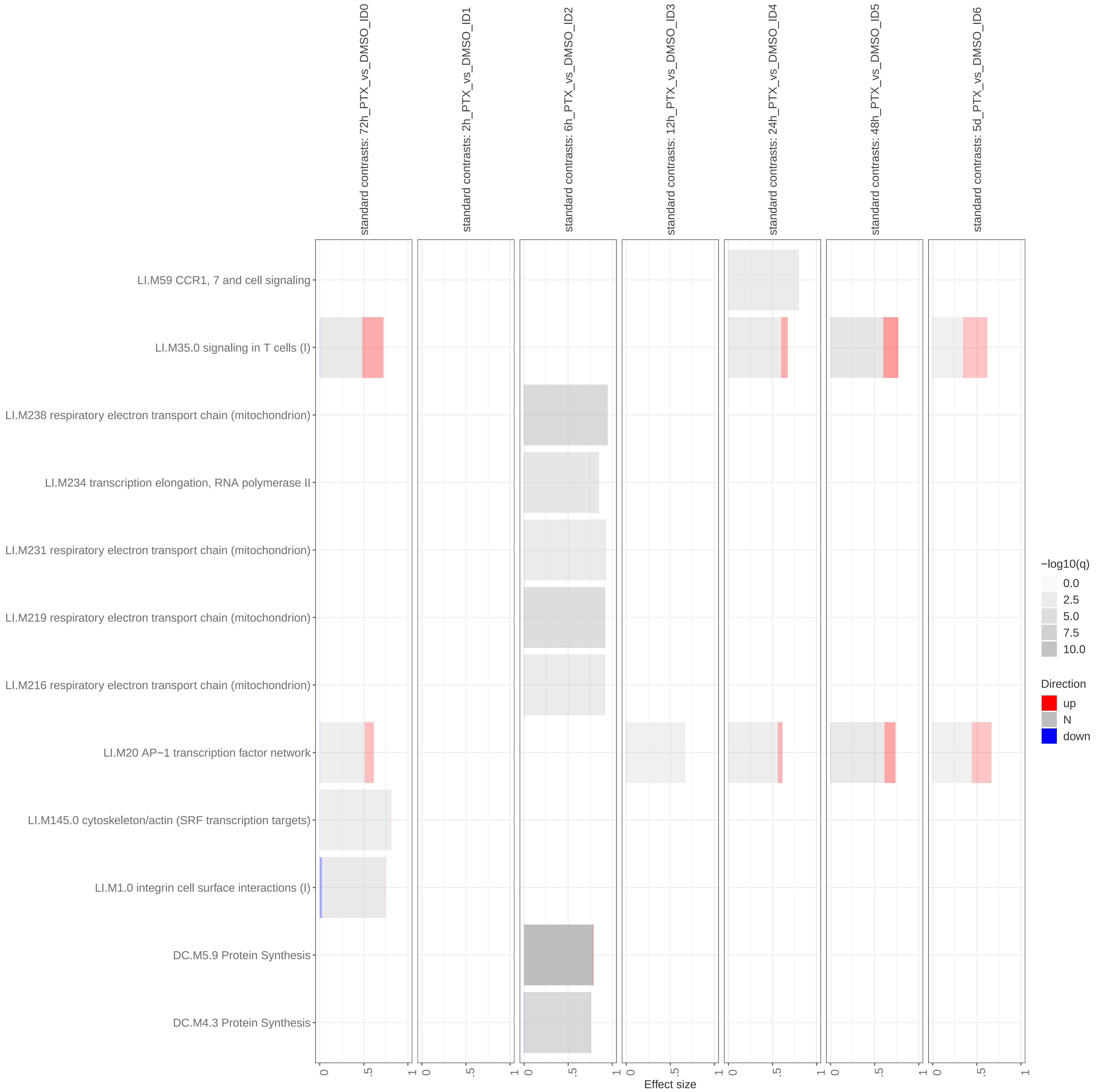
standard contrasts: 72h_PTX_vs_DMSO_ID0
standard contrasts: 2h_PTX_vs_DMSO_ID1
standard contrasts: 6h_PTX_vs_DMSO_ID2
standard contrasts: 12h_PTX_vs_DMSO_ID3
standard contrasts: 24h_PTX_vs_DMSO_ID4
standard contrasts: 48h_PTX_vs_DMSO_ID5
standard contrasts: 5d_PTX_vs_DMSO_ID6
LI.M59 CCR1, 7 and cell signaling
LI.M35.0 signaling in T cells (I)
LI.M238 respiratory electron transport chain (mitochondrion)
LI.M234 transcription elongation, RNA polymerase II
LI.M231 respiratory electron transport chain (mitochondrion)
LI.M219 respiratory electron transport chain (mitochondrion)
LI.M216 respiratory electron transport chain (mitochondrion)
LI.M20 AP−1 transcription factor network
LI.M145.0 cytoskeleton/actin (SRF transcription targets)
LI.M1.0 integrin cell surface interactions (I)
DC.M5.9 Protein Synthesis
DC.M4.3 Protein Synthesis
0
.5
1
Effect size
−log10(q)
0.0
2.5
5.0
7.5
10.0
Direction
up
N
down
